# Osteopontin plays a pivotal role in increasing severity of respiratory syncytial virus infection

**DOI:** 10.1371/journal.pone.0192709

**Published:** 2018-04-20

**Authors:** Viviana Sampayo-Escobar, Ryan Green, Michael B. Cheung, Raminder Bedi, Subhra Mohapatra, Shyam S. Mohapatra

**Affiliations:** 1 James A Haley Veterans Affairs Hospital, Tampa, Florida, United States of America; 2 Department of Internal Medicine, Department of Molecular Medicine, University of South Florida Morsani College of Medicine, Tampa, Florida, United States of America; 3 Department of Molecular Medicine, University of South Florida Morsani College of Medicine, Tampa, Florida, United States of America; Louisiana State University System, UNITED STATES

## Abstract

The molecular mechanisms underlying susceptibility to severe respiratory syncytial virus (RSV) infection remain poorly understood. Herein, we report on the role of osteopontin (OPN) in regulation of RSV infection in human epithelial cells and how interleukin-1 beta (IL-1β), a cytokine secreted soon after RSV infection, when persistently expressed can induce OPN expression leading to increased viral infection. We first compared OPN expression in two human epithelial cell lines: HEK-293 and HEp-2. In contrast to HEp-2, HEK-293 expresses low levels of pro-caspase-1 resulting in decreased IL-1β expression in response to RSV infection. We found a correlation between low IL-1β levels and a delay in induction of OPN expression in RSV-infected HEK-293 cells compared to HEp-2. This phenomenon could partially explain the high susceptibility of HEp-2 cells to RSV infection versus the moderate susceptibility of HEK-293 cells. Also, HEK-293 cells expressing low levels of pro-caspase-1 exhibit decreased IL-1β expression and delayed OPN expression in response to RSV infection. HEK-293 cells incubated with human rIL-1β showed a dose-dependent increase in OPN expression upon RSV infection. Also, incubation with rOPN increased RSV viral load. Moreover, HEp-2 cells or mice infected with a mucogenic RSV strain RSV-L19F showed elevated levels of OPN in contrast to mice infected with the laboratory RSV strain rA2. This correlated with elevated levels of OPN following infection with RSV-L19F compared to rA2. Together, these results demonstrate that increased OPN expression is regulated in part by IL-1β, and the interplay between IL-1β and OPN signaling may play a pivotal role in the spread of RSV infection.

## Introduction

Respiratory syncytial virus (RSV) is one of the most common causes of lower respiratory tract infections with a global disease burden estimated at ~34 million new cases and 160,000 deaths every year. RSV is one of the first pathogens encountered by the infant immune system and most infants have at least one RSV infection by two years of age. However, RSV may re-infect individuals throughout life because infection does not lead to a persistent immune memory response [1–5]. Healthy adults infected with RSV typically experience mild cold-like symptoms. However, severe RSV infection commonly causes bronchiolitis in infants resulting in 120,000 hospitalizations annually in the US. Severe RSV infection constitutes a high risk for the development of childhood asthma [6–8]. Elderly persons also develop severe RSV-induced pneumonia that leads to increased morbidity and mortality in this age group causing >11,000 deaths annually in the US alone [9, 10]. Despite progress made towards understanding the biology of RSV infection, the molecular mechanism which determines the severity of RSV infection is not well understood [11–13].

RSV infection induces a persistent inflammatory response which sometimes escalates beyond control [14–17]. This exaggerated inflammation complicates the disease outcome and leads to respiratory complications such as asthma exacerbation or recurrent wheezing, making it difficult to identify a treatment option [18–25]. Immune cells and tissues express pattern-recognition receptors (PRRs) capable of recognizing pathogen-associated molecular patterns (PAMPS), activating the innate immune response to release pro-inflammatory cytokines that facilitate pathogen clearance but also mediate disease pathology [26, 27]. RSV infection induces the expression of several pro inflammatory cytokines including IL-1β, IL6 and chemokines such as IL-8 and TNF-α that contribute to inflammation and the pathology of the infection. However, whether this inflammation contributes to increased viral load and spread of infection is unclear.

In an effort to dissect the molecular basis of severity of RSV infection, previously we conducted a microarray analysis and identified several genes whose expressions are influenced by both aging and RSV infection. Our previous studies in a murine model compared the progression of RSV infection in aged vs. young mice. We showed that aged mice express higher levels of IL-1β and OPN prior to infection compared to their younger counterparts, and this pro-inflammatory state that comes with aging impairs the antiviral response in those mice when they are exposed to RSV infection [28].

OPN is a secreted multifunctional protein also known as secreted phosphoprotein 1 (SPP-1) and early T-lymphocyte activation-1 (Eta-1) factor [29]. Of note, OPN expression is regulated by mediators of acute inflammation such as IL-1β [30]. Although it was first identified in osteoclasts and is highly expressed in bone, OPN is secreted by a variety of cells and tissues including macrophages, smooth muscle cells, epithelial, and endothelial cells [29, 31–34]. OPN can have both anti- and pro-inflammatory activities; hence it has been associated with many physiological functions and pathological conditions [35–39]. Integrin αvβ3 and CD44 are receptors for OPN and their interaction modulates the immune response by facilitating neutrophil and macrophage migration to sites of injury. Also, OPN promotes dendritic cell (DC) maturation and migration to the lymph nodes where DCs can present antigens to naïve T cells, thus serving as a bridge between the innate and the cell-mediated immune responses [33, 40–42].

Since OPN is up-regulated in other inflammatory lung diseases such as asthma and RSV has been implicated in asthma exacerbation, we reasoned that OPN might be involved in regulating susceptibility to RSV infection. To test this hypothesis, we investigated the role of OPN in RSV infection and determined that IL-1β expression correlated with the up-regulation of OPN during RSV infection. We also found increased OPN protein expression in human epithelial cells and mice infected with RSV-L19F, a strain that produces severe infection associated with increased viral loads, compared to rgRSV-A2 which induces mild infection and moderated viral loads. This suggests that OPN expression is correlated with increased spread of the virus between cells and increased cell permissiveness to RSV infection.

## Material and methods

### Mice

Female osteopontin-deficient knockout mice (OPN KO) (strain B6.Cg-Spp1tm1Blh/J) and wild type (WT) C57BL/6 were purchased from Jackson Laboratory and intranasally infected with RSV when they were 6–8 weeks old. All animal work was approved by and performed in accordance with the policies of the University of South Florida Institutional Animal Care and Use Committee. Mice were acclimated for 7 days prior to the start of experiments. Mice were provided with standard rodent chow and water ad libitum.

### Cell culture

Human epithelial type 2, HEp-2 (CCL 23; American Type Culture Collection, Rockville, MD), and human embryonic kidney 293, HEK-293 (ATCC CRL-1573), cells were maintained in Dulbecco´s modified Eagle´s medium (DMEM, HyClone) supplemented with 5% fetal bovine serum (FBS, HyClone) and 1% penicillin-streptomycin (GIBCO). Cells were incubated at 37°C in a humidified incubator with 5% CO_2_/95%.

### Virus purification, infection and plaque assay

Three different sources of RSV were used: rgRSV-A2 which expresses green fluorescent protein, the mucogenic rA2-L19F (RSV-L19F) recombinant variant of the A2 strain with its fusion protein replaced with that of the line 19 RSV, and a version of the line 19 that expresses a red fluorescent protein (RSV-KL19F). The virus was propagated by infecting 60% confluent HEp-2 cells with a multiplicity of infection (MOI) of 0.1 plaque forming units (pfu) per cell for two hours at 37°C with gentle rocking every 15 minutes, after which the medium was replaced by fresh DMEM containing 5% FBS. Cells and media were collected when 70–80% of the cells showed cytopathological effects. RSV was pelleted through a layer of 30% glycerol (0.22 μm-filtered) in 0.1 M MgSO_4_ and 50 mM HEPES, pH 7.5. The viral particles were pelleted by centrifuging at 11,600 rpm in an SW28 rotor for 3 h at 4°C. Supernatants were carefully aspirated without disturbing the viral pellets and the viral pellets were re-suspended in pre-cooled, 0.22 μm-filtered 50 mM HEPES, pH 7.5, 0.1 M MgSO_4_, 150 mM NaCl. This buffer was used as mock infection media in all experiments and the re-suspended pellet was aliquotted and stored at −80°C until use. UV-inactivation of RSV was performed by irradiating aliquots of virus with 1200 mJ of UV for 20 mins using a Stratalinker.

For all experiments, a monolayer of HEp-2 or HEK-293 cells at 80% confluence was infected with 0.1, 0.5, 1 or 10 MOI (as indicated in the figure legends). Cells were incubated with the viral inoculum in Opti-MEM (Life Technologies) containing 2% FBS for 2 hours at 37°C. After this, the infectious medium was replaced by fresh DMEM with 5% FBS. At 24, 48 or 72 hours post infection (hpi) the cell supernatants and pellets were collected for viral plaque assay, RNA or protein analysis.

WT or OPN KO mice were intranasally infected with 1 or 3 x 10^6^ plaque forming units (pfu)/mouse of RSV-L19F or rgRSV-A2 and euthanized 1, 3 or 5 days post-infection (dpi). Lungs were collected for RNA or protein extraction.

To determine the viral titers, HEp-2 cells were seeded in 24 well plates to 80% confluence and infected in duplicates with serial dilutions of cell supernatants for a period of 2 hours at 37°C. Infectious media were removed and cells were overlaid with 1 ml of 0.8% methylcellulose in DMEM supplemented with 5% FBS. 5 dpi, the cell monolayers were fixed overnight with 1 ml of 80% cold methanol. The next day, cells were rinsed with phosphate-buffered saline (PBS, Hyclone) and incubated with primary monoclonal antibody against RSV fusion protein (AbD Serotec). Plaques were visualized using an anti-mouse IgG horseradish peroxidase antibody (HRP)(Sigma) and developed with 4 CN peroxidase substrate (KPL); the dark purple spots were counted and each spot represented one plaque-forming unit (PFU).

### Quantitative RT-PCR

Total RNA was isolated from cells using TRIzol (Life Technologies). Samples were treated with recombinant DNase I (Life Technologies) to remove any contaminating DNA. 1 μg of RNA was reverse transcribed using Maxima First Strand cDNA Synthesis Kit for RT-qPCR (Thermo Fisher Scientific) as described in manufacturer’s instructions. Quantitative real-time PCR (qPCR) was performed on the BioRad CFX384™ Real-time PCR Detection system using DyNamo Color Flash SYBR master mix (Thermo Fisher Scientific). The sequences of all primers used in this study are as follows (forward and reverse): RSV-N, 5’- CAT CTA GCA AAT ACA CCA TCC A-3′ and 5′-TTC TGC ACA TCA TAA TTA GGA GTA TCA A-3′; human IL-1β (PrimeTime® -qPCR primers-IDT), 5’- GAA CAA GTC ATC CTC ATT GCC-3’ and 5’-CAG CCA ATC TTC ATT GCT CAA G-3’; human OPN, 5’-TGG CCG AGG TGA TAG TGT G-3’ and 5’- CGG GGA TGG CCT TGT ATG-3’; human IFN-β, 5’-CAA CTT GCT TGG ATT CCT ACA AAG-3’ and 5’- TGC CAC AGG AGC TTC TGA CA-3’. All samples were run in four replicates and the data were analyzed using normalized gene expression (ΔΔCt). Expression of all genes was normalized to control hypoxanthine-guanine phosphoribosyltransferase (HPRT): mouse HPRT, 5′-GCT GAC CTG CTG GAT TAC ATT AA-3′ and 5′-TGA TCA TTA CAG TAG CTC TTC AGT CTG A-3′; human HPRT, 5’- AGG AAA GCA AAG TCT GCA TTG TT-3’ and 5’- GGC TTT GTA TTT TGC TTT TCC A-3’.

### Western immunoassay

Cells were plated in 6-well culture plates the day before treatment/infection and harvested in lysis buffer containing 1% NP-40, 150 mM NaCl, 50 mM Tris-HCl pH 8.0 and a protease inhibitor cocktail (Thermo Fisher Scientific). After removal of cellular debris by centrifugation, total protein concentration was measured at 660nm using protein assay reagent (Thermo Fisher Scientific) and 25 μg of total protein were separated in a precast 12% mini-PROTEAN TGX gel (Bio-Rad) and transferred to a nitrocellulose membrane (Bio-Rad). The membrane was incubated with a rabbit polyclonal antibody to human OPN (Abcam, ab181440) and a mouse monoclonal antibody to β-actin (Sigma-Aldrich); proteins were detected by incubating with a secondary anti-mouse IgG-HRP and/or anti-rabbit IgG-HRP, followed by the ECL reagent kit (Pierce). Images were captured using a ChemiDoc XRS+ imaging system (Bio-Rad).

### Immunofluorescence microscopy

HEp-2 cells were plated in 8-well chamber slides the day before infection. Cells were mock infected or infected with 1 MOI of RSV strain RSV-L19F. Cells were fixed with cold 4% paraformaldehyde 24 or 48 hpi and stained with a goat polyclonal antibody against RSV (Millipore, ab1128) and a rabbit polyclonal antibody to OPN (ABCAM), followed by indirect immunofluorescence using secondary anti-goat IgG Alexa Fluor-555- and anti-rabbit IgG Alexa Fluor-488-conjugated antibodies. Slides were mounted with 4′, 6-diamidino-2-phenylindole (DAPI) containing anti-fade mounting media (Southern Biotech). A minimum of 10 images at 200X magnification were collected per slide with a DP72 digital camera on a BX51 Olympus fluorescence microscope on the three different fluorescence channels.

### Enzyme-linked immunosorbent assay (ELISA) for OPN and IL-1β in lung homogenates

Snap frozen lungs were homogenized in cold lysis buffer containing 10 mM Tris-HCl pH 8.0, 150 mM NaCl, 1% NP-40, 10% Glycerol, 5 mM EDTA and a protease inhibitor cocktail. Tissue debris was pelleted by centrifugation at 4°C for 10 min at 300×g and protein concentration in the supernatants was measured at 660 nm with protein assay reagent (Thermo Fisher Scientific). Mouse osteopontin ELISA kit (RayBiotech) and mouse IL-1β ELISA kit (BioLegend) were used per manufacturer’s instructions.

### Flow cytometry assay for CD44 and RSV infected cells

HEp-2 or HEK-293 cells were seeded on 6-well plates at an appropriate cell density to reach 70 to 80% confluence. On the day of the experiment, each set of cell cultures was infected with RSV-KL19F for 2 hours. 24 hpi, cells were washed with PBS, detached with accutase (Life Technologies) and stained with DAPI to determine cell viability, FITC mouse anti-human CD44 antibody (BD Bioscience) or isotype control antibody. The percentage of cells expressing GFP (CD44 +) or RFP (RSV +) in each cell culture was determined by flow cytometry (BD FACS Canto II). Data was analyzed using BD FACS diva software.

### Recombinant IL-1β and OPN treatment

HEK-293 cells were infected with 1 MOI of RSV-L19F as described previously. Afterwards, the infectious media was replaced by fresh DMEM 5% FBS containing different concentrations (0–1 and 10 ng/ml) of human rIL-1β (PeproTech). Similarly, HEK-293 were treated four hours before infection with different concentrations (0–1–10–50–100 and 200 ng/ml) of human rOPN (PeproTech) diluted in fresh DMEM 5% FBS. Cells were infected with RSV-L19F at 0.1 MOI, and two hours after infection the media was replaced by fresh DMEM 5% FBS containing rOPN. Protein and supernatants were collected for viral titering and western immunoassay.

### Caspase I inhibitor (Ac-YVAD-CHO) treatment

HEp-2 cells were treated with 10 μM of caspase I inhibitor (Sigma-Aldrich) or DMSO (vehicle control) the two hours preceding the infection. Afterwards, cells were infected with 1 MOI of RSV-L19F, and two hours later the infectious media was replaced by fresh media containing 10 μM of caspase I inhibitor or DMSO. Protein and RNA were isolated 24 hpi.

### CD44 receptor-neutralization and RSV infection

HEp-2 cells were seeded on 24-well plate the day before infection. On the infection day, cells were pre-treated at room temperature for 20 minutes with 10 μg broad spectrum rat-anti human CD44 antibody (clone A020) or normal rat IgG (control) (Millipore). After the pre-treatment, cells were infected with RSV-KL19F for two hours at 37°C and infectious media was replaced with fresh growth media. After 24 hours, the percentage of RSV positive cells was determined by flow cytometry (BD FACS Canto II). Data was analyzed using BD FACS diva software.

### Statistical analysis

All experiments were performed in triplicate and repeated at least twice. Statistical significance for each experiment was determined using Student’s t test and analysis of variance (ANOVA) with post-hoc Tukey’s multiple comparison test to find the differences among the groups, p<0.05. Calculations were performed and graphs produced using Prism 6.0 software (Graphpad Software, San Diego, CA, USA). Graphs of results show the mean and error bars depicting the standard error of the mean, +/- SEM.

## Results

### RSV infection upregulates IL-1β and OPN mRNA expression in epithelial cells

**Fig 1:**
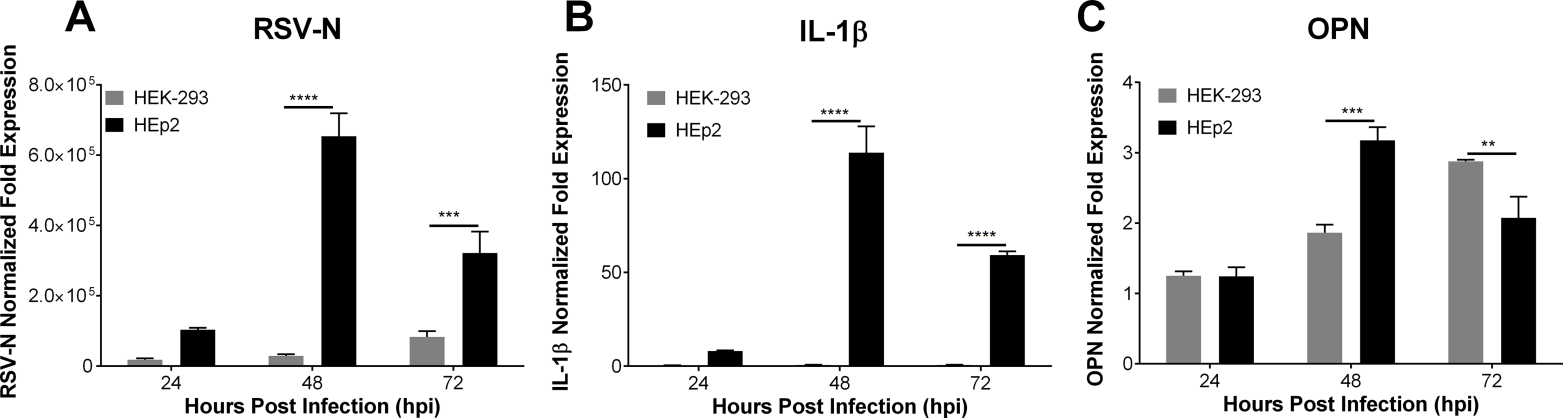
IL-1β and OPN mRNA expression in RSV infected cells. HEp-2 and HEK-293 cells were mock infected or infected with 1 MOI of RSV-L19F. RNA was isolated at 24, 48 and 72 hpi. (A-C) Expression levels of RSV-N, IL-1β and OPN were determined by qPCR. Results are presented as fold-change in expression of RSV-N, IL-1β or OPN mRNA normalized to the control (HPRT). qPCR data are represented as means ±SEM. Experiments were performed in triplicate. ** p < 0.01, *** p < 0.001**** p < 0.0001.

Our previous study in a murine model of infection showed that higher basal levels of IL-1β and OPN in aged mice are associated with impaired antiviral response that leads to increased susceptibility to RSV infection. *In vitro*, to evaluate the correlation of IL-1β and OPN expression with susceptibility to RSV infection we examined RSV infection in commonly used and highly susceptible HEp-2 cells and HEK-293 cells. HEK-293 cells were selected because they express low levels of endogenous pro-caspase1, which is essential for IL-1β expression; therefore, they constitute a good model to compare viral infection and OPN expression in diminished IL-1β expression [43]. We found a significant increase in expression (reported as fold expression compared to mock-infected cells) of RSV-N and IL-1β transcripts 24, 48 and 72 hpi in RSV-L19F-infected HEp-2 cells compared to RSV-L19F-infected HEK-293 cells (Fig 1A and 1B). We compared OPN expression levels obtained by qPCR performed on RNA extracted from HEp2 and HEK-293 cells infected with 1 MOI of RSV-L19F. In a similar pattern to that of IL-1β, OPN expression was significantly increased 48 hpi in RSV-L19F-infected HEp-2 cells. In contrast, HEK-293 cells showed decreased RSV infection, lack of IL-1β expression, and delayed OPN mRNA expression that was up-regulated 72 hpi (Fig 1C). These results suggest that increased RSV infection up-regulates both OPN and IL-1β expression. Also, lack of IL-1β expression in HEK-293 cells correlates with resistance to RSV infection and delays OPN expression.

### RSV infection-induced IL-1β regulates OPN expression

**Fig 2:**
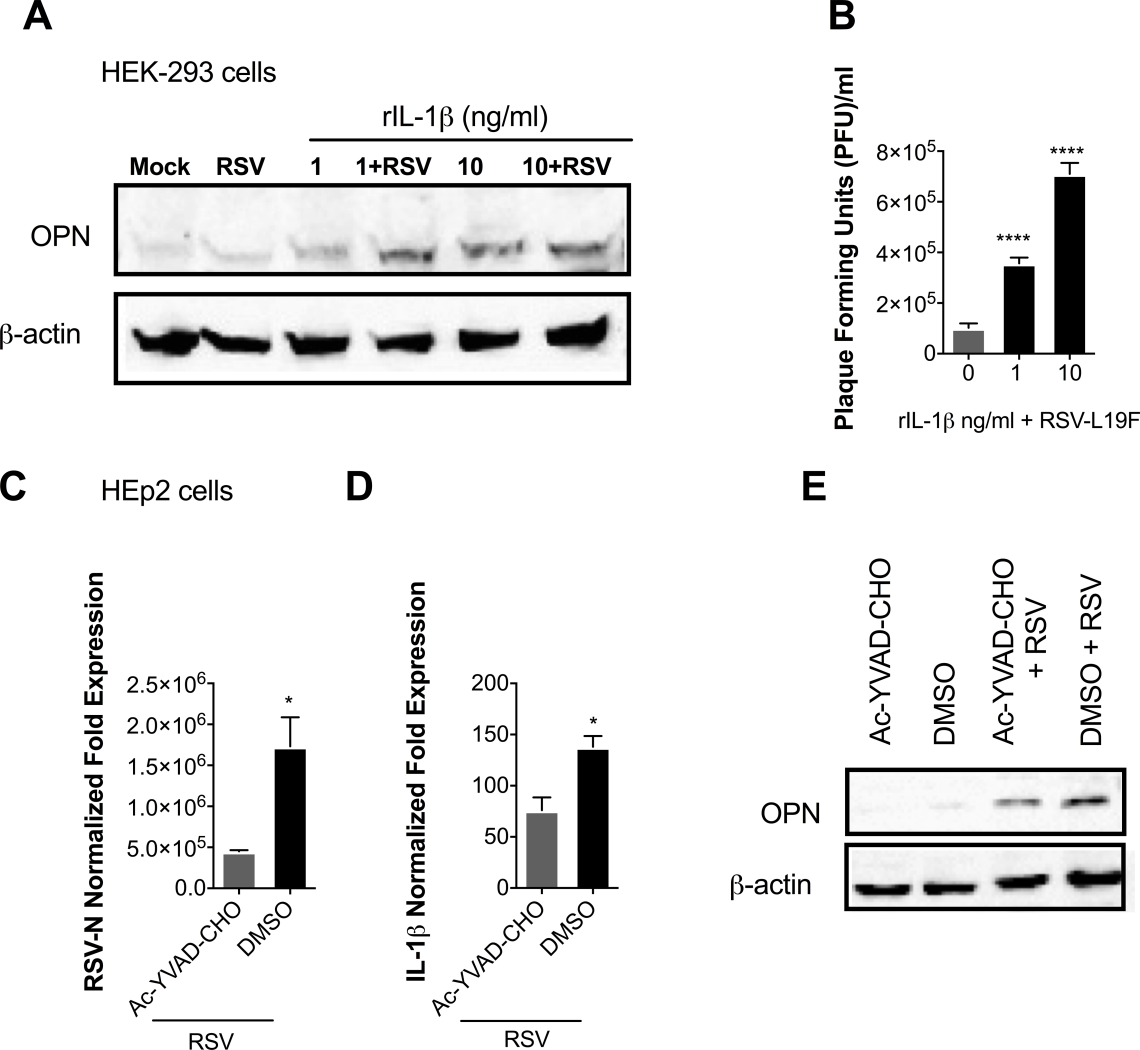
RSV induced IL-1β regulates OPN expression during RSV infection. (A-B) HEK-293 cells were mock-infected or infected with 1 MOI of RSV-L19F. 2 hours after the infection cells were treated with increasing concentrations of human rIL-1β (0, 1 or 10 ng/ml). (A) Protein was harvested 24 hpi for western blots. 25μg of protein lysate was loaded in each lane. OPN is seen at 55 kDa and β-actin (loading control) at 42 kDa. (B) Plaque viral titers were obtained from the supernatants collected 24 hpi. (C-E) HEp-2 cells were pre-treated two hours prior to RSV-L19F infection with 10 μM of Ac-YVAD-CHO (caspase-I) inhibitor. Cells were infected with 1 MOI of RSV-L19F and infectious media was replaced with fresh media containing 10 μM of Ac-YVAD-CHO. RNA and protein were collected 24 hpi. (C and D) Expression levels of IL-1β and RSV-N were determined by qPCR. Results are presented as fold-change in expression of IL-1β or RSV-N mRNA normalized to the control (HPRT). (E) 25μg of protein lysate was loaded in each lane. OPN is seen at 55 kDa and β-actin (loading control) at 42 kDa. qPCR data are represented as means ±SEM. Experiments were performed in triplicate. **** p < 0.0001.

While the regulation of OPN expression is not fully understood, it is known that the expression of OPN is regulated by different stimuli, depending on the cells and tissues where it is expressed [40, 41, 44]. To examine the role of IL-1β in the induction of OPN expression and to confirm that the production of OPN is not intrinsically impaired in HEK-293 cells, cells were infected with 1 MOI of RSV-L19F, then the infectious medium was replaced with growth medium containing 1 or 10 ng/ml of human rIL-1β. Results showed that there was no OPN expression with RSV infection without rIL-1β treatment; however, incubation of HEK-293 cells with human rIL-1β led to increased OPN expression 24 hours after treatment. In addition, the RSV-L19F-infected HEK-293 cells treated with rIL-1β showed higher levels of OPN expression compared to uninfected cells treated only with rIL-1β (Fig 2A). While rIL-1β increased the expression of OPN after mock or RSV-L19F infection, it also contributed to an increase in viral yield in RSV-infected cells (Fig 2B). Since HEK-293 and HEp-2 cells are likely to have other genetic differences which may influence OPN expression or susceptibility to RSV infection, we treated HEp-2 cells with a caspase-1 inhibitor (Ac-YVAD-CHO) in order to mimic the reduced IL-1β expression seen in HEK-293 and establish that IL-1β is responsible for the observed differences in RSV infection and OPN expression between the two cell lines. Cells were treated with the inhibitor and subsequently infected with RSV-L19F (1 MOI). Our results showed that cells treated with caspase-I inhibitor showed decreased RSV-N and IL-1β mRNA levels (Fig 2C and 2D). Also, OPN protein expression was significantly reduced in those cells treated with the inhibitor as compared to control group (Fig 2E). These results demonstrate the regulatory effect of IL-1β and OPN expression in the progression of RSV infection.

### RSV replication is not required for increased OPN expression

**Fig 3:**
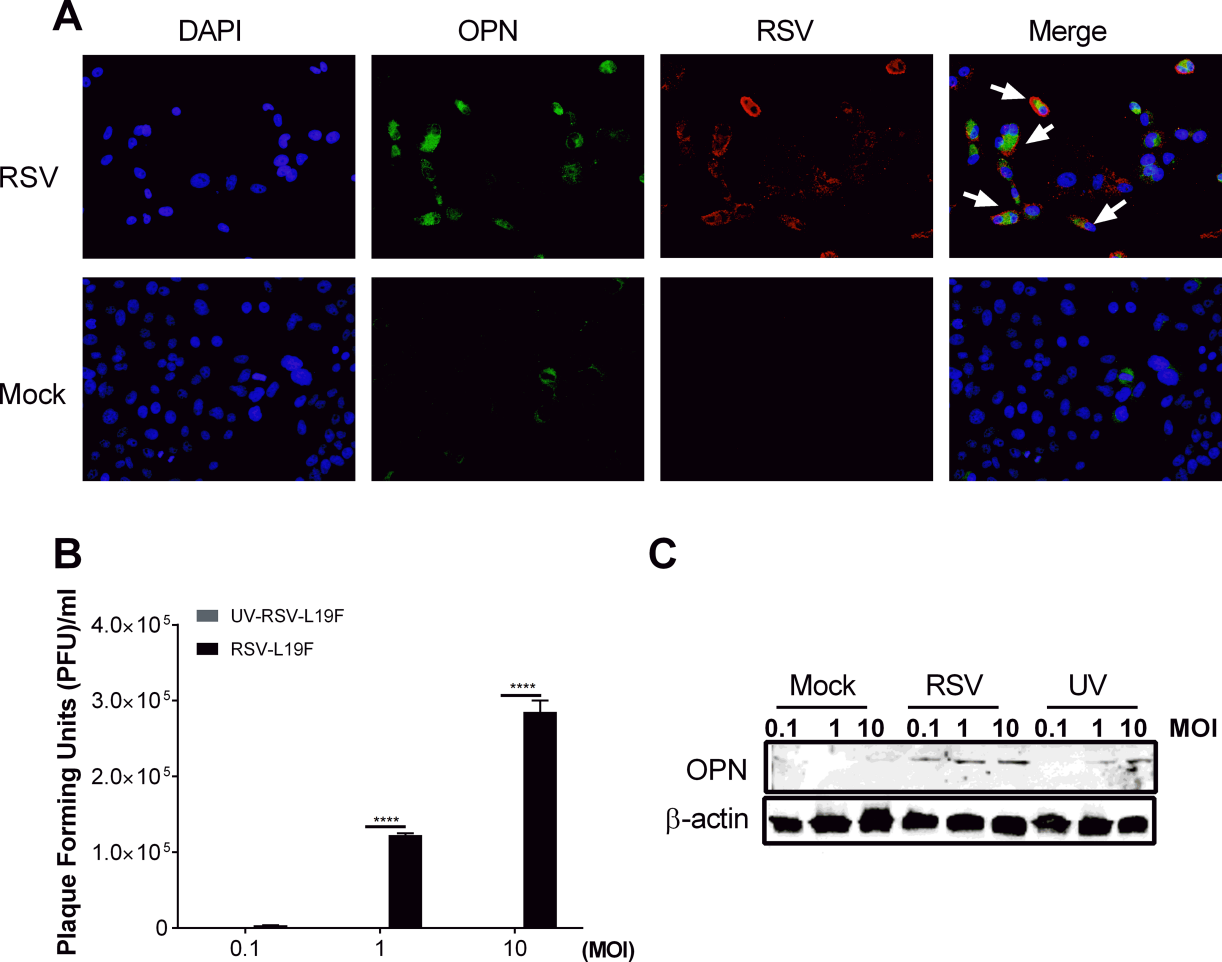
RSV infection induces OPN expression in a dose dependent manner. HEp-2 cells were mock-infected or infected with RSV-L19F (1 MOI). (A) Images of Hep-2 cells stained with polyclonal antibody against RSV and OPN at 48 hpi. Representative images of RSV positive (red) cells, OPN positive (green) cells, and DAPI (blue) nuclear staining at 200X magnification. (B) RSV titer by plaque assay of supernatants for increasing doses (0.1, 1 and 10 MOI) of UV-inactivated and native RSV-L19F at 48 hpi. (C) OPN protein (55 kDa) expression in HEp-2 cells at 48 hpi after mock, RSV and UV-inactivated RSV treatment in the indicated doses analyzed by western blots. β-actin (loading control) at 42 kDa was probed as the loading control.

To assess baseline levels of OPN and subsequent changes in expression upon RSV infection, HEp-2 cells were infected with RSV-L19F at 1 MOI (1 PFU/cell) and the levels of OPN were confirmed by immunostaining of mock- or RSV-L19F-infected HEp2 cells; results showed elevated OPN expression in the virus-infected cells compared to mock-infected cells. There were comparatively fewer OPN-positive cells in the mock-infected than RSV-L19F-infected cells 48 hpi (Fig 3A).

To examine the role of RSV replication during OPN induction, HEp-2 cells were infected with increasing doses (0.1, 1 and 10 MOI) of UV-inactivated RSV-L19F, native RSV-L19F or mock treatment. To evaluate the productivity of the infection, supernatants were used for RSV titration by plaque assay. As expected, the number of PFU increased in a dose-dependent manner from 0.1 to 10 MOI in RSV-L19F infected cells. There was no productive infection in the UV-attenuated virus (Fig 3B). The cells were lysed at 48 hpi for Western blot analyses. OPN expression was proportional to the MOI dose of the RSV—higher in 10 MOI-infected cells compared to 0.1 or 1 MOI-infected cells (Fig 3C). Furthermore, OPN expression was higher in RSV-L19F-infected cells than in UV-inactivated infected cells; however, at 10 MOI of UV-inactivated RSV there was up-regulation in the expression of OPN suggesting that some RSV proteins or nucleic acids in the inactivated form of RSV continued to contribute to OPN induction and expression.

### rOPN increases RSV titers in a dose-dependent manner

**Fig 4:**
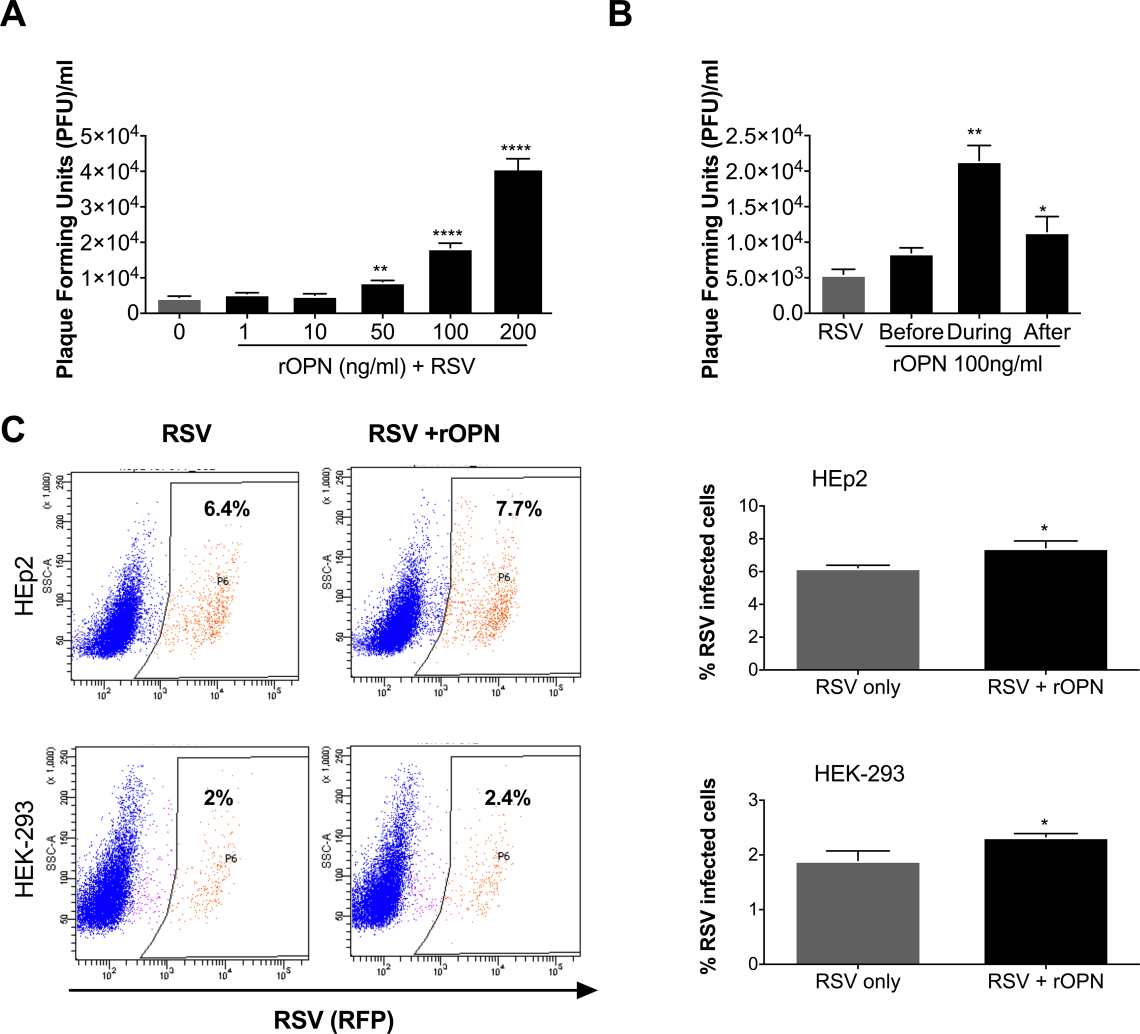
Human rOPN increases RSV titers in a dose-dependent manner. (A) HEK-293 cells were treated with increasing concentrations of human recombinant OPN (rOPN) (0 to 200 ng/ml) four hours before infection. Cells were infected with 0.1 MOI of RSV-L19F and infectious media was replaced with fresh media containing the appropriate concentration of rOPN. Supernatants were collected 24 hpi and viral titers were determined by plaque assay. (B) HEK-293 cells were treated with 100 ng/ml at different time points: before, during or immediately after the infection. Supernatants were collected 24 hpi and viral titers were determined by plaque assay. Result of a representative experiment is shown. * p < 0.05, ** p < 0.01, **** p < 0.0001. (C) Hep-2 or HEK-293 cells were infected with 0.1 MOI of RSV-KL19F or RSV-KL19F in the presence of 100 ng/ml of rOPN. After 24 hours, the infected HEp-2 or HEK-293 cells were gated for RFP-expression (RSV + cells).

To better understand the effect of increased OPN expression on viral yield during RSV infection and to validate that OPN expression is sufficient for the increase in viral titer seen in HEK-293 cells, we used varying concentrations of human rOPN (0, -1, 10, -50, 100 and 200 ng/ml) to pretreat HEK-293 cells four hours before infection with 0.1 MOI of RSV-L19F. After infection, the medium was replaced with fresh growth medium containing rOPN at the same concentrations. 24 hours after infection, there was a significant dose-dependent increase in viral titers in cells treated with rOPN, further confirming that OPN promotes RSV infection (Fig 4A). Additionally, to determine the effects of exogenous rOPN in modulating RSV infection, an intermediate OPN concentration (100 ng/ml) known to cause an effect on RSV infection was selected to treat the cells at three different time points: to one group of cells, rOPN was added to the media four hours before the infection, later the media was aspirated and cells were rinsed before infection; in the second group, rOPN was added during the two hours of infection, then media was aspirated from the cells and fresh media was added; in the third group, after the two hours of infection, the media was aspirated and replaced with fresh media containing rOPN. Our results show a significant increase in viral titers of cells treated with human rOPN during or after the infection (Fig 4B). As an additional test of rOPN effect during the infection, HEp-2 and HEK-293 cells were infected with 0.1 MOI of the clinical strain Line 19 that expresses a red fluorescent protein-RFP (RSV-KL19F) in the presence of rOPN. We evaluated the number of RSV infected cells using flow cytometry. We found that treatment with rOPN significantly increased the percentage of infected HEp-2 and HEK-293 cells by 23% and 20% respectively (Fig 4C). These findings suggest that OPN could facilitate the entry of the virus into the cells and increase cell permissiveness to RSV infection.

### CD44 modulates RSV infection

**Fig 5:**
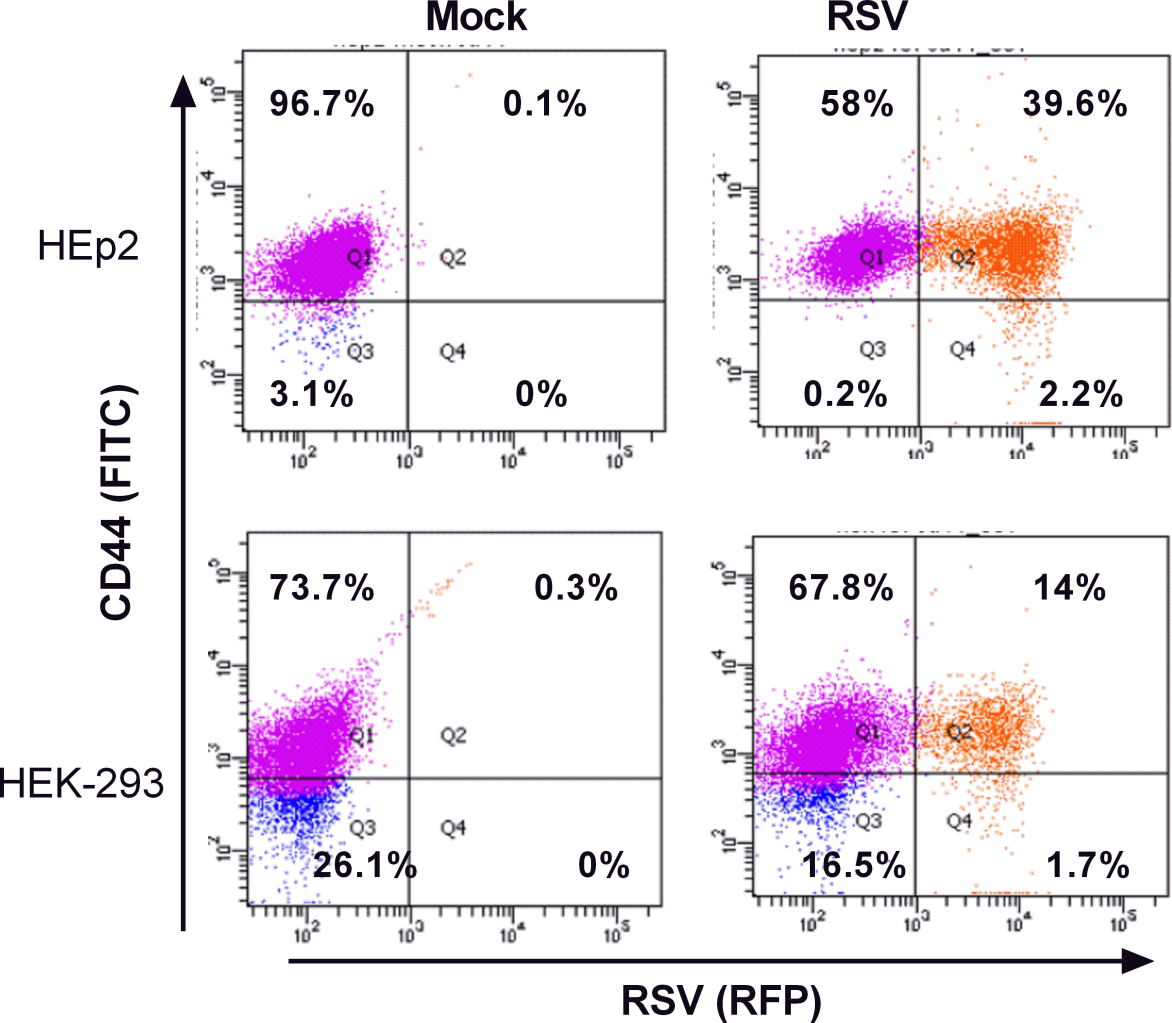
CD44 expression mediates RSV infection. HEp-2 or HEK-293 cells were infected with 0.5 MOI of RSV-KL19F (red). After 24 hours, the cells were stained with FITC mouse anti-human CD44 antibody (green) and gated for RSV expression (red). (Top panel) Flow cytometry analysis of HEp-2 cells infected with RSV-KL19F. (Bottom panel) Flow cytometry analysis of HEK-293 cells infected with RSV-KL19F. Result of a representative experiment is shown. Experiments were performed in triplicate.

CD44 is known to be a receptor for OPN. To evaluate the expression of CD44 in mock or RSV infected cells, HEp-2 and HEK-293 cells were infected with 0.5 MOI of RSV-KL19F. 24 hours after infection, cells were stained with a FITC-CD44 antibody and gated for CD44 (green) and RSV (red) expression. We found higher cell surface expression of CD44 in the HEp-2 cell line (96.7%) than in HEK-293 (73.7%). We also found a significant difference in the number of RSV positive cells confirming that HEp-2 is more permissive to RSV infection (Fig 5). These results suggest that the earlier OPN expression in HEp-2 cells and the concomitant CD44 expression could be key mediators for OPN signaling during RSV infection that potentiates the infection of HEp-2 cells.

**Fig 6:**
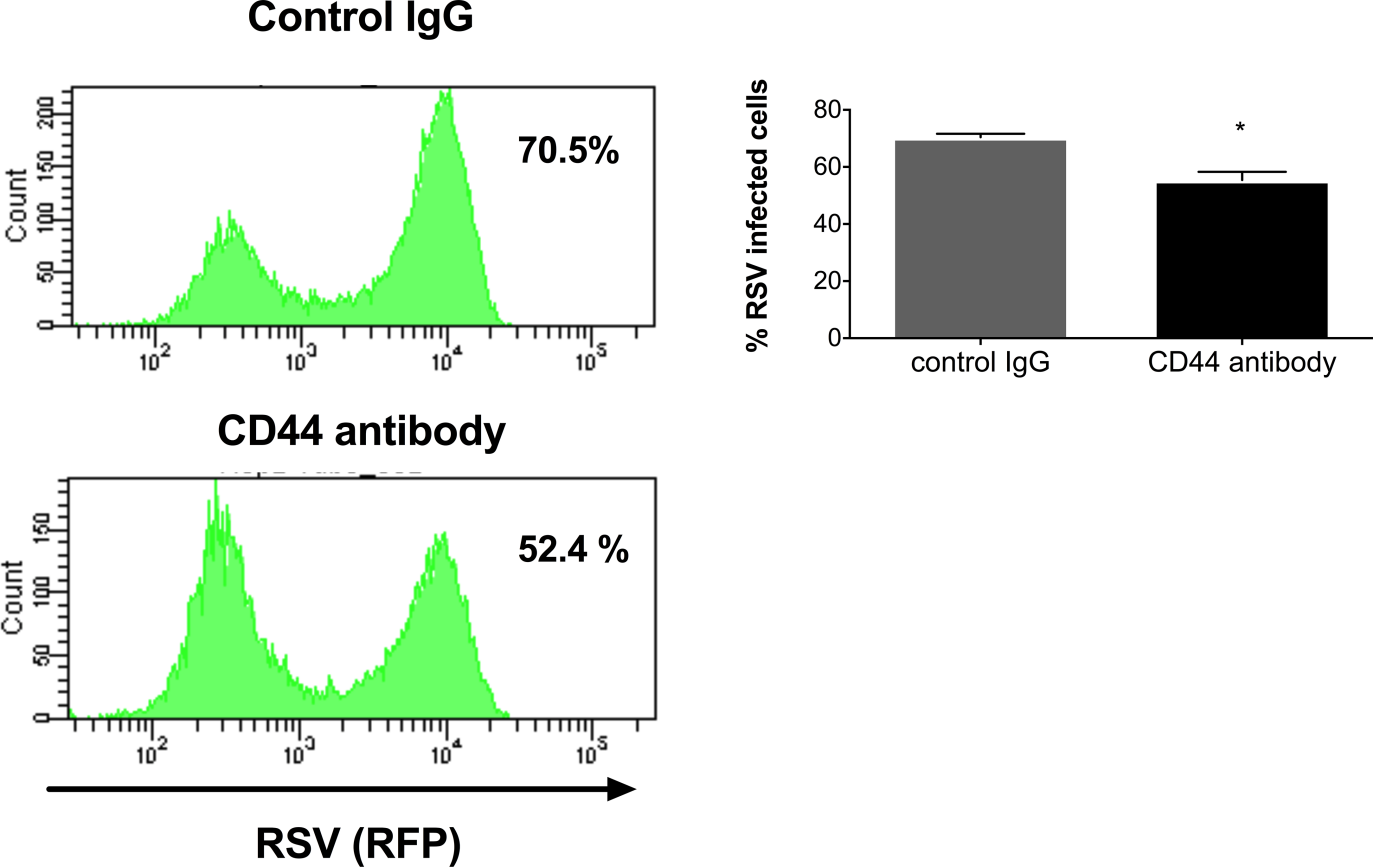
Neutralization of CD44 receptor reduces the number of RSV positive cells. Hep-2 cells were pre-treated with 10 μg of broad spectrum rat-anti human CD44 antibody (clone A020) or normal rat IgG (control). After the pre-treatment, cells were infected with RSV-KL19F (0.5 MOI) and infectious media was replaced with fresh growth media. After 24 hours, the percentage of RSV positive cells was determined by flow cytometry. Result of a representative experiment is shown. Experiments were performed in triplicate.

To test whether CD44 expression is required for RSV infection, the CD44 receptor was neutralized with a broad-spectrum rat-anti human CD44 antibody. Briefly, HEp-2 cells were pre-incubated during 20 minutes at room temperature and then infected with RSV-KL19F (0.5 MOI) for two hours, after which the infectious media was replaced with fresh growth media. 24 hpi we assessed the number of RSV positive cells (RFP +) by flow cytometry. We found ~ 52.4% of the cells were RSV positive in the cells pre-treated with CD44 antibody while there was 70.5% RSV positive cells in the control group. Our results showed a decrease in RSV positive cells following treatment with anti-CD44 antibody prior to infection compared to normal rat IgG control (Fig 6). Together, these data suggest that CD44 may be involved in facilitating the RSV infection process.

### OPN expression is a marker of high RSV loads

**Fig 7:**
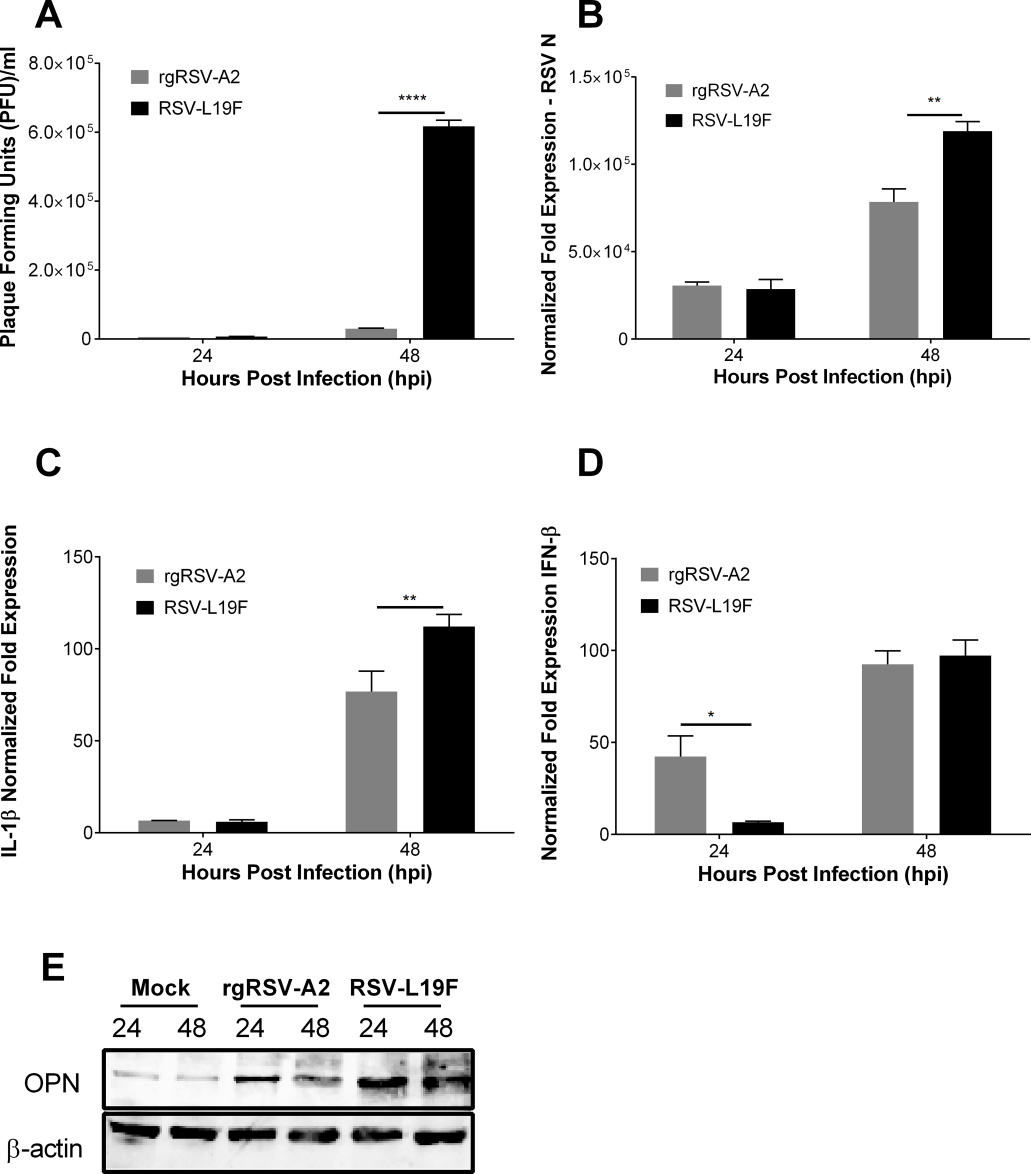
OPN is a marker of high RSV loads in HEp-2 cells. HEp-2 cells were mock-infected or infected with 1 MOI of RSV-L19F or rgRSV-A2. RNA, supernatants and protein were isolated 24 and 48 hpi. (A) Plaque titers were obtained from supernatants of HEp-2 cells infected with RSV-L19F or rgRSV-A2. (B—D) Expression of RSV-N, IL-1β and IFN-β were determined by qPCR. Results are presented as fold-change in expression of RSV-N, IL-1β or IFN-β mRNA normalized to the control (HPRT). (E) Western blot analysis of OPN expression. 25μg of protein lysate was loaded in each lane. OPN is seen at 55 kDa and β-actin (loading control) at 42 kDa. Experiments were performed in triplicate. * p < 0.05, ** p < 0.01, **** p < 0.0001.

To investigate the role of OPN in determining the severity of RSV infection, HEp-2 cells were infected with rgRSV-A2 or RSV-L19F. The latter is known to induce severe RSV infection and higher viral loads in a mouse model [45]. *In vitro*, infection with rgRSV-A2 and RSV-L19F resulted in similar plaque viral titers and RSV-N gene expression at 24 hpi (Fig 7A and 7B). However, at 48 hpi RSV-L19F-infected cells had a significantly increased number of viral plaques and a similar increase in RSV-N mRNA expression when compared to cells infected with rgRSV-A2 (Fig 7A and 7B). Following the same pattern, we found no significant difference in the gene expression of IL-1β at 24 hpi with the two RSV strains, but we noted a significant up-regulation of IL-1β mRNA expression 48 hpi with RSV-L19F compared to rgRSV-A2 (Fig 7C). Remarkably, IFN-β mRNA levels were significantly elevated 24 hpi in rgRSV-A2-infected cells compared to those infected with RSV-L19F. This may in part account for the difference in viral titers and RSV-N transcripts observed in cells infected with the different viral strains (Fig 7D). Western blot analysis revealed an increase in OPN protein expression at 24 hpi that remained high at 48 hpi in RSV-L19F-infected cells. Cells infected with rgRSV-A2 showed less OPN protein expression at 24 hpi and the expression started to return to baseline levels by 48 hpi (Fig 7E).

### RSV replication is diminished in mice lacking OPN (OPN KO)

**Fig 8:**
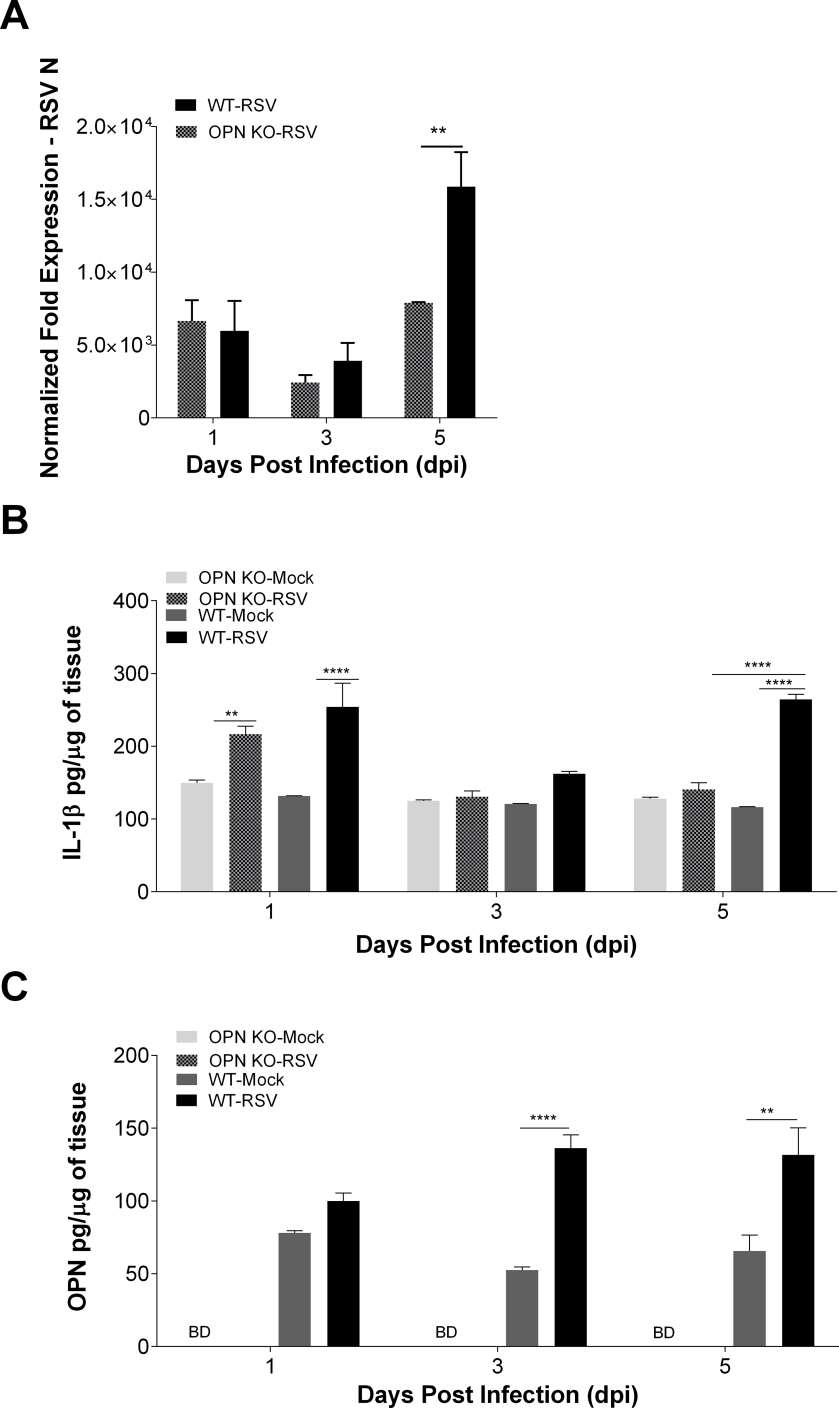
RSV replication is diminished in mice lacking OPN (OPN KO). C57BL/6 and OPN KO mice were mock-infected or infected with 3 x 10^6^ RSV-L19F. Lungs were collected 1, 3 and 5 dpi for protein and RNA extraction. (A) RSV-N mRNA expression levels were determined by qPCR and results represented as fold-change in expression of RSV-N mRNA normalized to the control (HPRT). (B and C) Levels of OPN and IL-1β in lung homogenates were determined by ELISA. BD: Below detection limits. Lung homogenates were obtained from individual mice and not pooled (n>4 per group). ** p < 0.01, **** p < 0.0001.

To determine the time points where IL-1β and OPN expression are induced and their effect during RSV infection, we infected OPN KO and WT mice intranasally with the mucogenic virus, RSV-L19F. Mice were euthanized 1, 3 and 5 dpi. As we reported before, RSV infection resolves much faster in OPN KO mice than WT. This was evidenced by a significant decrease in RSV-N transcripts at 5 dpi in the lungs of OPN KO mice as measured by qRT-PCR. There were no significant differences in RSV-N amplification 1 or 3 dpi, but the infection did not progress to 5 dpi in OPN KO mice as it did in WT mice (Fig 8A). Secretion of IL-1β was also measured upon infection since it was suspected to play a role in controlling OPN expression during RSV infection. We found a significant increase in IL-1β expression 1 dpi in both OPN KO and WT mice infected with RSV-L19F; yet there was no significant difference between the two strains of mice at 1 or 3 dpi. Nonetheless, at 5 dpi RSV infected WT mice had increased levels of IL-1β while in the OPN KO mice IL-1β levels returned to baseline (Fig 8B). The expression of OPN in RSV-L19F-infected WT mice was also determined and compared to mock-infected WT. OPN protein levels were measured by ELISA of lung homogenates. We observed a significant increase in OPN protein levels at 3 and 5 dpi in WT mice infected with L19F (Fig 8C). As expected, the levels of OPN were below the detection limit in RSV-L19F or mock-infected OPN KO mice (Fig 8C).

**Fig 9:**
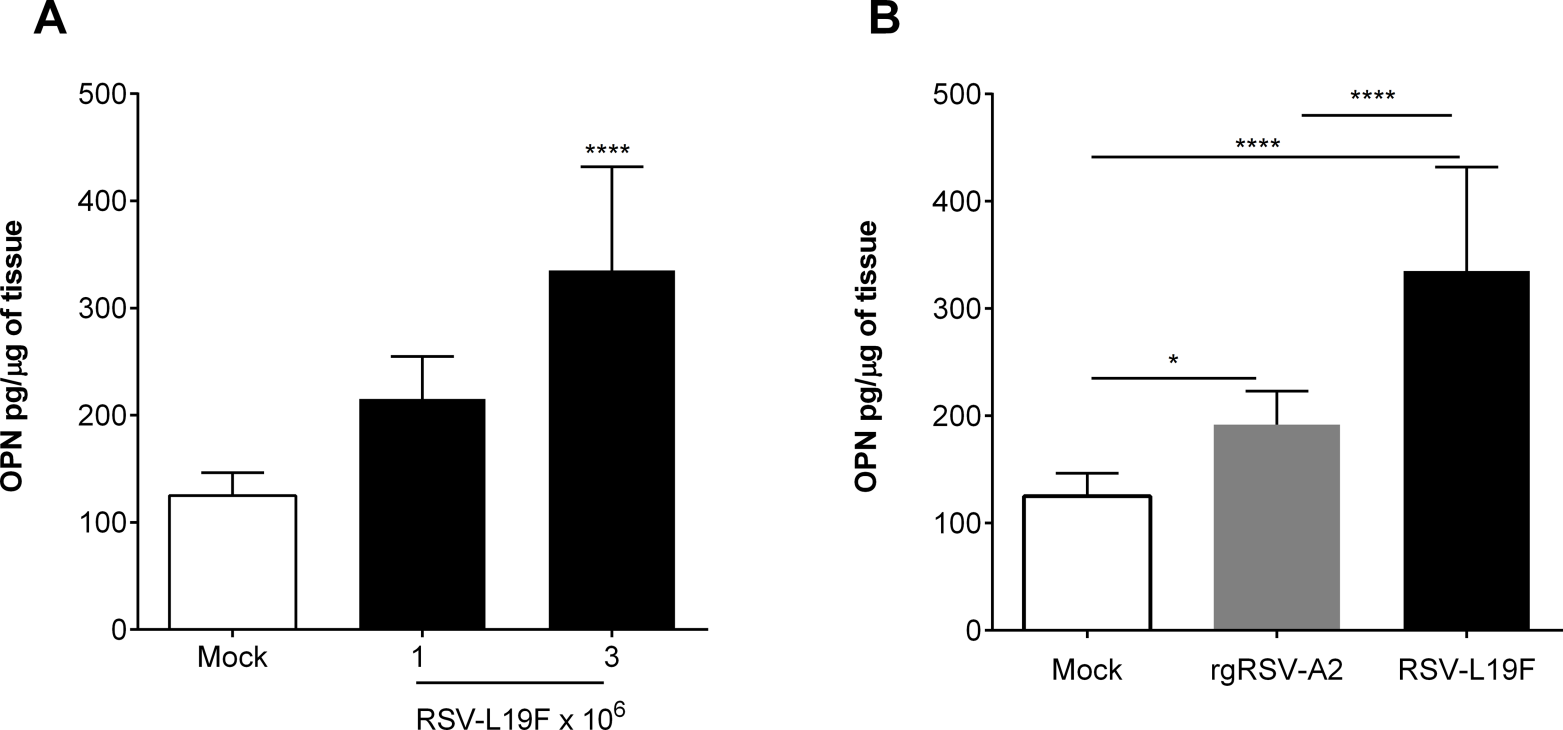
OPN expression is a marker of high RSV loads *in vivo*. (A) C57BL/6 mice were mock-infected or infected with 1 or 3 x 10^6^ RSV-L19F. (B) C57BL/6 mice were mock-infected or infected with 3 x 10^6^ RSV-L19F or rgRSV-A2. (A-B) Lungs were collected 3 dpi for protein isolation and levels of OPN in lung homogenates were determined by ELISA. Lung homogenates were obtained from individual mice and not pooled (n>4 per group). * p < 0.05, ** p < 0.01, **** p < 0.0001.

Furthermore, we infected WT mice in increased doses of RSV-L19F (1 or 3 x 10^6^) and found a significant increase in OPN protein levels that correlated with the increased viral concentration used to infect those mice (Fig 9A). We also evaluated the effect of mild or severe RSV infection *in vivo* and how it influenced OPN expression. WT mice were infected with mock, RSV-L19F or rgRSV-A2. Similar to the results found *in vitro*, WT mice infected with RSV-L19F exhibited higher OPN protein levels compared to those infected with rgRSV-A2 (Fig 9B). These results suggest that increases in OPN expression levels are tightly associated with RSV viral loads.

## Discussion and conclusions

To our knowledge our lab was the first to determine the importance of OPN during RSV infection, and the results from our *in vivo* and *in vitro* experiments in the present study show how OPN expression is linked with increased susceptibility to RSV infection. The salient findings of the present studies are as follows: i) RSV infection leads to increased IL-1β and OPN expression, ii) IL-1β is involved in the regulation of OPN levels during RSV infection, iii) OPN on its own can enhance RSV infection and contribute to viral spread, and iii) OPN is a predictive marker of viral loads both *in vitro* and *in vivo*.

A major finding of our study is that IL-1β is involved in up-regulating OPN expression levels during RSV infection in HEp-2 cells, but the same OPN up-regulation is not observed in HEK-293 cells which has disrupted expression of IL-1β. Our results suggest that OPN plays an important role in RSV infection and propagation *in vivo* and *in vitro*, and IL-1β amplifies the inflammatory response by inducing OPN expression, thus resulting in increased viral loads. Our results show that although RSV infection can induce significant OPN expression in the absence of IL-1β, in the presence of IL-1β it can enhance OPN expression and accelerate the infection process.

The induction of OPN is controlled by a variety of cytokines, growth factors and hormones [40, 46, 47]. A previous study showed that IL-1β dramatically increased OPN expression during pulmonary fibrosis through the activation of ERK1/2 but not by JNK pathway [30, 48]. Likewise IL-1β is one of the factors released at sites of injury that contributes to enhanced OPN expression [49]. RSV infection induces a substantial increase of IL-1β, a pro-inflammatory cytokine known to induce the expression of a plethora of downstream pro-inflammatory cytokines including OPN, thus resulting in magnification of the inflammatory process [43, 50–52]. We have explored the association between IL-1β and OPN up-regulation during RSV infection and our data shows that OPN and IL-1β expression leads to increased viral infection. We also show a delay in OPN mRNA expression levels in infected HEK-293 cells compared to HEp-2 cells, suggesting that the impaired production of IL-1β in HEK-293 cells partially contributes to the reduced expression of OPN in the infected HEK-293. The increased early OPN expression in HEK-293 cells treated with rIL-1β proves that the production of OPN is not intrinsically impaired in HEK-293 cells but instead is dependent on IL-1β signaling. The correlation between increased OPN protein levels and increased RSV viral titers in HEK-293 cells treated with rIL-1β further confirms the role of these two pro-inflammatory cytokines in the regulation of RSV infection. Similarly, to rule out other differences between HEK-293 and HEp-2 cells, we inhibited caspase-I expression in HEp-2 cells and showed that this inhibition leads to a decrease in IL-1β expression that also results in decreased OPN expression and decreased viral infection.

In addition, we have found that OPN on its own can enhance RSV infection. OPN can have anti- and pro-inflammatory activity; hence it has been associated with physiological and pathological conditions [47, 53]. To establish the mechanism for OPN up-regulation and also to verify that OPN was expressed in human epithelial cells infected with RSV, we infected HEp-2 cells with RSV-L19F and verified through immunostaining, Western blotting and qPCR that RSV triggers OPN expression. The result that infected cells were found to express more OPN indicates that RSV infection per se is a major trigger of OPN induction. Also, we found up-regulation of OPN after infection with UV irradiated-RSV-L19F, while the UV inactivated virus led to lower expression of OPN protein levels compared to replicating RSV-L19F. We tested if UV—RSV would similarly affect IL-1β response and we did not find such a response, suggesting that RSV replication was needed for the IL-1β response (S1 Fig). This data also suggests that OPN is partially regulated by IL-1β during RSV infection. These results indicate that loading more viral proteins and/or nucleic acids potentiates OPN induction even in the absence of viral replication and suggest that viral replication is required for the overexpression of OPN. Nonetheless, in the absence of RSV replication a small increase in OPN was observed, which may be attributed to the ssRNA induced innate immune response.

We have consistently observed an increase in OPN expression in cells and mice infected with RSV. In order to evaluate the feedback effect of high OPN levels on RSV infection we treated HEK-293 with human rOPN during RSV infection. Our results showed that HEK-293 cells treated with human rOPN displayed a dose-dependent increase in viral titers, suggesting that OPN has a regulatory effect on RSV infection. Moreover, the results of studies on timing of OPN action show the prominent effect of OPN during the infection process itself. Cells treated with rOPN during and immediately after the infection yielded higher viral titers, while cells pre-treated with OPN before the infection did not show a significant difference in viral titers when compared to RSV infected cells without rOPN treatment. This suggests that OPN mediates increased entry and/or assembly of the virus leading to a significant increase in plaque viral titers.

Further, cells treated with rOPN became infected with RSV at a significantly higher rate than untreated cells. Also, HEp-2 highly expressing the OPN receptor CD44 showed a higher percent of infected cells compared to HEK-293 cells whose expression of CD44 is lower. Although the basis of OPN regulation of RSV entry and/or assembly remains unclear, one possibility is that OPN modulates the fusion process itself, thus aiding virus entry. This idea is supported by the observation that the OPN receptor (CD44) co-localizes with RSV F protein and results in viral filament formation and syncytia formation which benefit the infectious process [54]. Additionally, the decrease we observed in RSV-positive cells after CD44 receptor neutralization evidenced that any manipulation of components of the lipid rafts which disrupts CD44 signaling could negatively affect the infection process; thus our results suggest that the interaction of proteins at the lipid rafts, like OPN and its receptor (CD44), could favor the infection process. Also, CD44 is expressed in a variety of immune cells, thus it is likely that the interaction of OPN-CD44 during RSV infection may play a role in modulating both innate and the adaptive immune response, which in turn plays a role in RSV clearance.

Moreover, we found that OPN is a predictive marker of severe RSV infection. A comparative analysis of virus infection, IL-1β, OPN and IFN-β expression by two RSV strains (lab isolate rA2 versus a mucogenic strain RSV-L19F) showed that OPN expression was significantly higher in RSV-L19F compared to rA2 infected cells. Also, our *in vivo* studies in a mouse model of RSV infection showed that IL-1β expression preceded OPN expression in WT mice after RSV infection. Importantly, we found a significant difference in infection at 5 dpi, where WT infected mice exhibited increases in RSV-N gene amplification compared to OPN KO mice. This data is consistent with a previous study done by our lab where we found decreased viral titers from lung homogenates and fewer RSV-positive lung cells in the OPN KO mice infected with RSV compared to WT mice [28]. Further, these results are consistent with reports of increased levels of OPN positively correlated with severity of lung inflammation [55–59]. OPN up-regulation in serum and tissue samples from chronic rhinosinusitis and allergic patients also correlated with severity of disease [55, 60, 61]. In agreement with these studies, a recent paper found a positive correlation between increased levels of OPN in human serum and disease severity in influenza patients [62]. Our *in vitro* finding suggests that IFN-β reduces the viral infection resulting in decreased IL-1 β and OPN expression and is consistent with previous reports where RSV-A2 infection resulted in significantly higher expression of IFN-α than RSV-L19F in human epithelial cells and BALB/c mice [45, 63]. Additional studies have reported decreased OPN levels in serum samples from multiple sclerosis patients treated with IFN-β, supporting this IFN-β dependent mechanism of OPN regulation [64–66]. Also, an inverse relationship between IFN-β and OPN expression reported in recent data indicates that the fusion protein of the virus could directly modulate type I IFN expression and that the increase in viral loads after RSV-L19F infection lead to higher expression levels of viral non-structural proteins (NS1 and NS2) that are known to disrupt type I IFN response and therefore may act to increase OPN [45, 67–69]. Together, these pathophysiological differences between non-severe and severe RSV strains suggest that OPN could be used as a marker of severe RSV infection.

**Fig 10:**
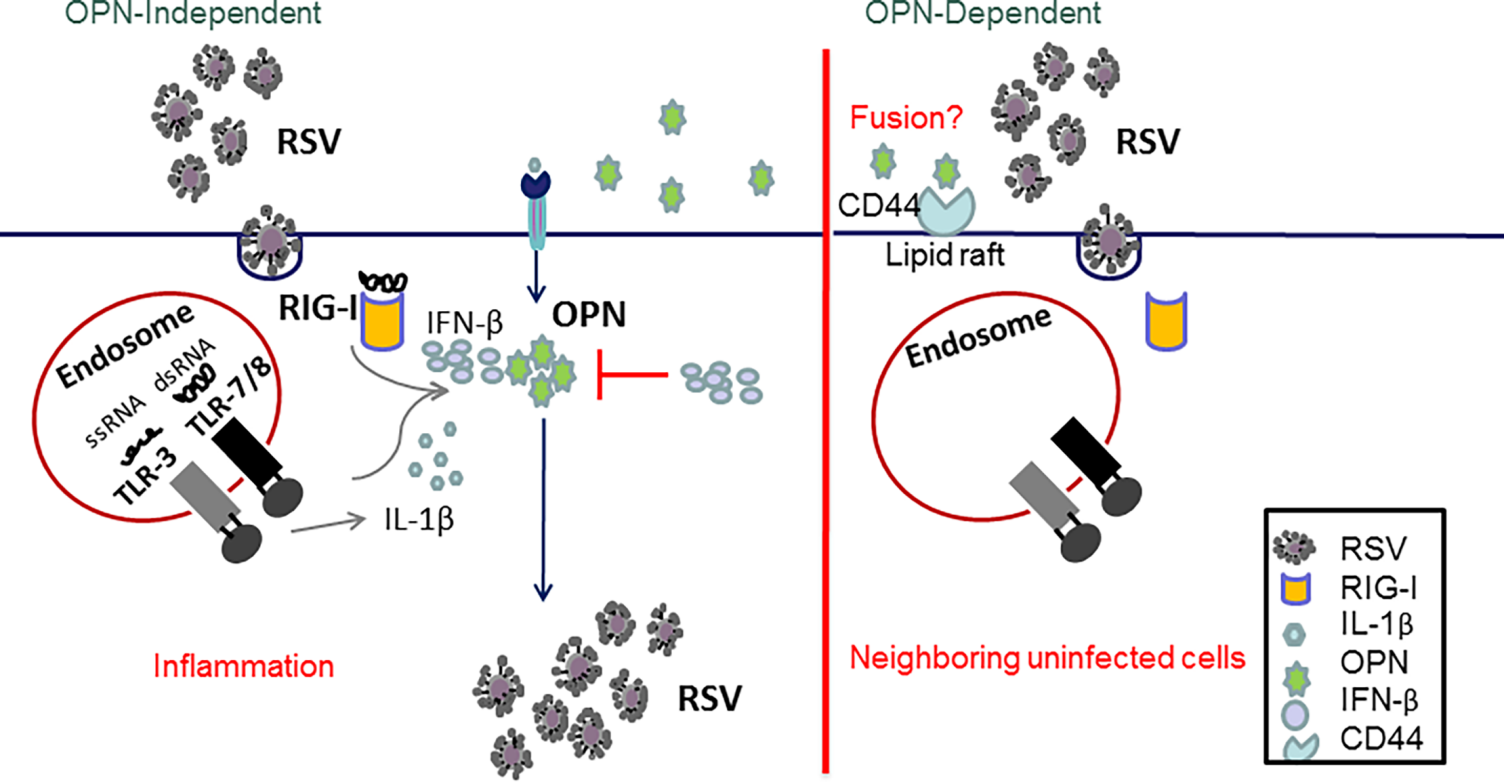
Model for the role of OPN during RSV infection. (Left panel) OPN-independent model. During RSV infection, the virus is recognized by the host cells through PRR (TLR-3, RIG-I, TLR-7); recognition of the virus leads to activation of innate immune response which leads to release of anti-viral and pro-inflammatory cytokines like IFN-β and IL-1β. Expression of IL-1β leads to up-regulation of OPN whereas expression of IFN-β down-regulates OPN expression. (Right panel) OPN-dependent model. Fusion of RSV is accelerated in the presence of OPN and its receptor (CD44), thus leading to increased number of infected cells which results in increased viral loads.

Proper control of the inflammatory response is required not only for effective viral clearance but also for prevention of subsequent complications caused by imbalanced immune response and exaggerated inflammation during RSV infection [70]. It has been suggested that the severity of the disease caused by RSV is not only controlled by replication of the virus but also by the host inflammatory response. Therefore, these two factors together could help us predict the severity of the disease and perhaps establish new treatments aiming to control infection and inflammation [71–73]. An accurate profiling of inflammatory mediators is a promising strategy to explore RSV physiopathology that could contribute to a better management of RSV disease. Together, the results of these studies have pointed to the role of OPN during the first cycle of RSV infection (OPN-independent cycle), where the viral nucleic acid is recognized by PRR, thus leading to IL-1β expression and subsequently to OPN up-regulation. Therefore, a second replication cycle of the virus would be OPN-dependent; the interaction of OPN and its receptor (CD44) would result in an increased viral fusion to subsequent host cells that could result in increased number of infected cells and therefore viral production (Fig 10).

In conclusion, our studies suggest that IL-1β positively regulates OPN expression in the context of RSV infection. We also recognize that there could be other contributing factors like viral proteins or other cytokines found up-regulated after RSV infection that will control OPN expression. Also, increased levels of OPN protein are sufficient to increase viral loads, thus influencing the onset and severity of infection. Thus, we show that OPN could be used as a marker of severe infection or higher RSV loads. The knowledge acquired from this research could be used in the future to develop new drug targets for treatment and/or prophylaxis of RSV infection since it may lead to development of pharmacological strategies that allow for regulation of OPN expression during infection.

## Supporting information

S1 Fig IL-1β mRNA expression in UV-inactivated or native RSV-L19F HEp-2 infected cells. HEp-2 cells were infected with 1 MOI of UV-irradiated or native RSV-L19F. RNA was isolated at 24, 48 and 72 hpi. Expression levels of IL-1β were determined by qPCR. Results are presented as fold-change in expression of IL-1β mRNA normalized to the control (HPRT).
(DOCX)
