# Supplementary material for: Osteopontin plays a pivotal role in increasing severity of respiratory syncytial virus infection
Source: PLoS One. 2018 Apr 20;13(4):e0192709. doi: 10.1371/journal.pone.0192709 (PMC5909912; doi:10.1371/journal.pone.0192709)

**S1 Fig. IL-1β mRNA expression in UV-inactivated or native RSV-L19F HEp-2 infected cells.**HEp-2 cells were infected with 1 MOI of  UV-irradiated or native RSV-L19F. RNA was isolated at 24, 48 and 72 hpi. Expression levels of IL-1β were determined by qPCR. Results are presented as fold-change in expression of IL-1β mRNA normalized to the control (HPRT).


Supplement: S1 Fig — HEp-2 cells were infected with 1 MOI of UV-irradiated or native RSV-L19F. RNA was isolated at 24, 48 and 72 hpi. Expression levels of IL-1β were determined by qPCR. Results are presented as fold-change in expression of IL-1β mRNA normalized to the control (HPRT). (DOCX) [file pone.0192709.s001.docx]
